# Alternative Water Transport and Storage Containers: Assessing Sustained Use of the PackH_2_O in Rural Haiti

**DOI:** 10.4269/ajtmh.18-0228

**Published:** 2019-03-04

**Authors:** Andrea L. Martinsen, Erin Hulland, Raina Phillips, Jean Allain Darius, Erica Felker-Kantor, Dan Simpson, Mariana Stephens, Evan Thomas, Rob Quick, Thomas Handzel

**Affiliations:** 1 Emergency Response and Recovery Branch, Division of Global Health Protection, Center for Global Health, Centers for Disease Control and Prevention, Atlanta, Georgia;; 2 Centers for Disease Control and Prevention, Haiti Office, Port-au-Prince, Haiti;; 3 Habitat for Humanity International, Atlanta, Georgia;; 4 Mortenson Center in Global Engineering, University of Colorado, Boulder, Colorado;; 5 Division of Foodborne, Waterborne, and Environmental Disease, National Center for Emerging and Zoonotic Infectious Diseases, Centers for Disease Control and Prevention, Atlanta, Georgia

## Abstract

The PackH_2_O water backpack carrier was developed to provide safe storage and relieve stress of head-loading during water transport with traditional containers such as buckets and jerry cans. We conducted an evaluation to assess both self-reported and observed use over a 6-month period between November 2014 and May 2015. A total of 866 packs were distributed to 618 households in six communities in rural Haiti, and 431 and 441 households were surveyed at midline and end line, respectively. We performed linear regression to assess change of self-reported use over time. Although 79.3% of respondents reported continued use of the 20-L pack after 6 months, other measures of self-reported use were low, with only 16.8% reporting to have used the pack the last time they collected water and 10.3% preferring the pack over other water collection containers. In addition, only 10.2% of all people collecting water at community sources were observed using packs and 12.0% of all households surveyed had water in the pack at the time of visit. Pack use varied by community and demographics. Although women were targeted during distribution, men preferred the pack and were more commonly observed using it at the community water sources. In conclusion, the use of the PackH_2_O was not widely adopted in rural Haiti; however, further research is needed to assess the pack acceptance in areas where back-loading is more common and in emergency settings.

## INTRODUCTION

Water collection is both a physical and time burden when not available on household premises, and women and children have primary responsibility of collection in almost three quarters of global households.^1–4^ Global access to basic drinking water services, defined as improved sources requiring no more than 30 minutes to collect, increased from 81.1% in 2000 to 88.5% in 2015. Despite this progress, an estimated 263 million people still have only limited access and spend more than 30 minutes per round trip to collect drinking water from improved water sources. Furthermore, unimproved sources, such as surface water and unprotected springs, are often located farther from home than improved sources.^5^

In a study of 24 sub-Saharan African countries, the proportion of rural households who traveled more than 30 minutes varied from 2% in Liberia to 58% in Mauritania. In 13 of the 24 countries, between 20% and 50% of rural households spent more than 30 minutes per trip to collect water.^3^ In Artibonite, Haiti, 29.6% of households reported their drinking water source to be more than 30 minutes away. Similar to that in other geographic areas, women were predominately responsible for water collection in Artibonite, with 81.1% of households reporting an adult female to be involved in water collection.^6^

Water collection containers are often carried on the head, although other methods of transport may include back-loading, rolling filled containers, or using animals.^7^ The existing literature suggests that carrying water on the head leads to back and neck pain and possible degenerative changes in the spine, given the weight of the loads and the repetitive stress.^7^ Whereas head-loading was found to be associated with neck pain, back-loading was associated with more areas of pain and discomfort than head-loading.^8^ However, both acute and long-term consequences of carrying water, either on the head or on the back, remain largely unknown.^7,8^ Typical containers for transporting and storing water include buckets or jerry cans, which are plastic, narrow-mouthed containers. These containers vary in size and may hold up to 20 L, weighing 44 pounds when filled with water. In Haiti, head-loading is common and the majority of households (89.1%) use a 20-L bucket as their primary storage container.^6^

**Figure 1.**
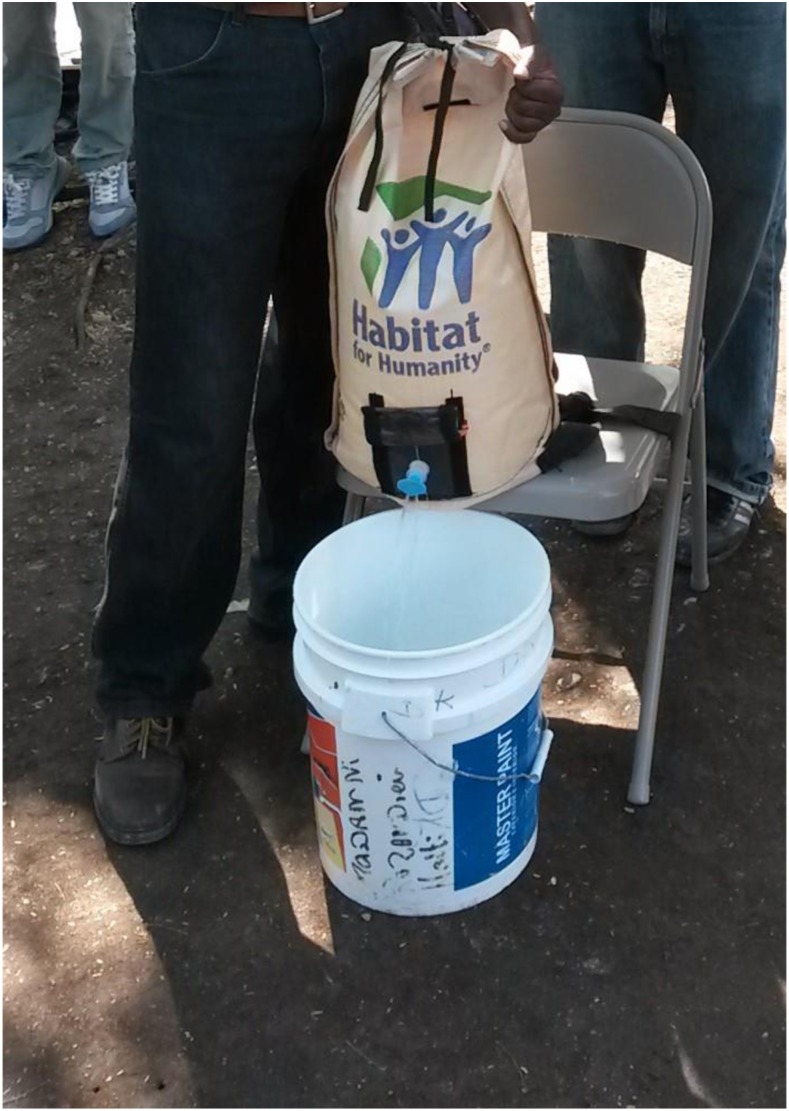
20-L PackH_2_O filled with water. This figure appears in color at www.ajtmh.org.

The PackH_2_O is a water backpack carrier, designed as an alternative to commonly used buckets or jerry cans to relieve the physical stress of carrying water on the head and to provide safe storage. It consists of a collapsible pack with a separate plastic liner that can be removed and easily cleaned and replaced if needed. The plastic liner has a spigot to dispense water directly from the pack. The pack comes in both 20- and 10-L sizes, which are intended for adults and children, respectively. The PackH_2_O is compact and lightweight to facilitate transport and distribution.^9,10^ The 20-L pack is shown in Figure 1.

Preliminary pilot evaluations of the PackH_2_O in Guatemala, Haiti, and Kenya suggested an improvement over traditional water collection and storage methods, including ease of transport and shortened water collection time. Pack acceptability was high; however, no independent evaluation of the PackH_2_O had previously been conducted.^11–13^ Habitat for Humanity collaborated with the manufacturer to distribute PackH_2_Os in both emergency and nonemergency settings. Before scaling up distributions, they requested the U.S. CDC to conduct two independent evaluations of the PackH_2_O where distributions were underway in Haiti and Kenya to assess the use and acceptability of the packs.

Herein, we present findings on the use and acceptability in rural Haiti. CDC researchers partnered with the Hôpital Albert Schweitzer (HAS), a local nongovernmental organization serving the Artibonite region. The HAS service area is largely rural and mountainous, and coverage of improved water sources in communities surrounding the hospital is limited. Habitat for Humanity provided support to the distribution of the PackH_2_Os, training on use, and the evaluation. The overall objective was to document the sustained use over a 6-month period by the target population in Haiti and to identify facilitators and barriers to use that influence uptake of the intervention.

## METHODS

### Population and pack distribution.

Six communities were conveniently selected from the HAS service area to receive packs based on a priori knowledge of population and water source type. To evaluate pack use under different conditions, HAS selected both rural and peri-urban communities with different types of water sources and with varying distance to primary water sources from community. The total estimated population of these six communities combined was 1,200. Leaders in each of the six communities were informed of the distribution and were instructed to invite all households within their communities to receive a pack. Only those households who sent a family member older than the age of 18 to scheduled distributions received a pack. All households with a family member present received a 20-L pack, and a subset of households with children, selected randomly but not systematically by partners, received 10-L packs because of limited availability. Habitat for Humanity and HAS staff were responsible for distribution and training of community members on use. They trained recipients on the use of the pack; how to fill, close, and carry the pack; and how to clean the liner. In total, 618 registered households received packs, including 613 who received 20-L packs and 253 who received 10-L packs.

### Data collection.

The evaluation took place between November 2014 and May 2015 and used a mixed-methods approach, using 1) cross-sectional surveys of pack beneficiaries at months 0, 3, and 6, 2) monthly monitoring of pack use at months 1, 2, 4, and 5, 3) observations at selected community water points at months 3 and 6, and 4) electronic sensor monitoring.

Cross-sectional surveys were conducted at three time points: the baseline at the point of distribution, a midline at the household after 3 months, and the end line at the household after 6 months. All households in the study area were registered at the point of distribution and systematically selected to survey at baseline. We selected households from the registration list using simple random sampling for midline and end line. The sample size of households was calculated based on an estimated expected use of 50% and 5% margin of error, yielding a sample size of 384. We oversampled by 20% to account for loss to follow-up and difficulty reaching all households, resulting in a final sample size at baseline of 480 households across all six communities. Clustering by community was not accounted for in sampling, but stratification by community was performed in the analysis. Data were directly collected using Samsung Galaxy tablets with the Open Data Kit (ODK, University of Washington, Seattle, WA) and aggregated using the ODK Briefcase and Microsoft Excel (Microsoft Corp., Redmond, WA). In addition to questions on water collection and storage practices, enumerators also observed the packs at the time of survey for the presence of stored water.

Community health workers conducted monthly monitoring of use during months where baseline, midline, and end line surveys did not occur (months 1, 2, 4, and 5) using paper-based forms on a subset of randomly selected households. Data were then entered into Microsoft Excel.

Objective measures of pack use, including water point observations and remote sensor monitoring, were carried out to complement the self-reported data from beneficiaries. During water point observations conducted at midline and end line, monitors were stationed at the primary water sources in five the six communities during peak collection hours in the morning and afternoon on two separate days. At each point, they used a data collection tool to record demographics of water collector, including an estimation of age, mode of transport, and type and quantity of containers.

CDC also collaborated with the Sustainable Water, Energy, and Environmental Technologies Laboratory (SweetSense Inc., Denver, CO) at Portland State University to embed electronic sensors in a systematically selected subset of distributed PackH_2_Os. More information on the methods and results can be found in supplemental files.

### Data analysis.

Pack use was assessed using multiple analytic methods evaluating self-reported and observed use over 6 months. We assessed self-reported use using three different proxy variables: 1) did the respondent still use the pack, 2) which container (or containers) was used during the last trip to collect water, and 3) preferred container for collecting water. We assessed observed use for transport as the proportion of people observed with either a 20-L or 10-L pack during water point observations of the total number of people observed. Observed use for storage was assessed by the proportion of households with water in the pack at the time of the visit.

We performed linear regression to assess change over time in self-reported use and used Chi-square analyses and Fisher’s exact tests (where expected cell counts were less than five) to determine whether there was a significant difference in both self-reported and observed use when stratified by community separately at midline and end line. Observed pack use for transport was stratified by age group and gender to account for any confounding variables. We assessed changes in water storage practice during the household visits via McNemar’s test. All analyses were conducted in SAS version 9.3 (SAS Institute, Cary, NC), and tables and figures were created with Excel and R.

The assessment protocol, including all survey tools, was approved by the CDC Institutional Review Board and the National Bioethics Committee of Haiti’s Ministry of Public Health.

## RESULTS

Of the 618 registered households from six communities, 613 received a 20-L pack, 253 households received a 10-L pack in addition to the 20-L pack, and five households received only a 10-L pack. In three of the six communities, approximately 100% of the total estimated households were reached. In Achen and Anger, 40% and 72% of households received the packs, respectively. Finally, in Vielot, 25% of the total population received packs; however, only one geographic area was targeted. The number of surveys administered in each of the six communities was proportional to the number of PackH_2_Os distributed, with a roughly equal proportion of surveys to packs distributed in each community (Table 1). Surveys were administered predominately to female household members (87.0%), as they were the primary targeted recipients of PackH_2_Os.

**Table 1:** Selected communities for PackH_2_O distributions in Artibonite department, Haiti, November 2014

Community	Achen	Anger	Champion	Mahoue	Marotte	Savan Bourg*	Vielot	Total
Type of community	Peri-urban	Rural	Rural	Rural	Rural	–	Rural	
Population (estimated no. of households)†	352	116	60	99	45	–	574	1,246
No. of households registered at distribution (% of total registered)	143 (23.1)	83 (13.4)	63 (10.2)	112 (18.1)	46 (7.4)	26 (4.2)	145 (23.5)	618
No. of 20-L packs distributed (% of total distributed)	143 (23.3)	82 (13.4)	63 (10.3)	109 (17.7)	46 (7.5)	26 (4.2)	144 (23.5)	613‡
No. of 10-L packs distributed (% of total distributed)	32 (12.6)	36 (14.2)	20 (7.9)	55 (21.7)	29 (11.5)	6 (2.4)	75 (29.6)	253‡
Surveys administered, *n* (% of total 20-L packs distributed)								
Baseline	91	70	49	91	42	10	87	440 (71.8)
Month 1	64	40	30	51	30	–	41	256 (41.8)
Month 2	66	45	32	52	33	–	52	280 (45.7)
Midline	104	64	51	93	40	2	77	431 (70.3)
Month 4	74	45	29	49	34	–	51	282 (46.0)
Month 5	73	45	28	49	35	–	50	280 (45.7)
End line	116	68	52	85	41	–	79	441** (71.9)

HAS = Hôpital Albert Schweitzer.

* Packs in Savan Bourg were distributed at Achen. No data available on type of community or estimated number of households. These households were excluded in analyses stratified by community but included in all aggregate analyses.

† Data provided by HAS.

‡ The number of actual packs distributed is likely to be much higher than 866, as the HAS staff and community health workers (CHWs) were overwhelmed in some of the communities and not able to register all beneficiaries.

** Excludes one observation from “other.”

Information was collected at baseline to assess typical household customs around water collection, storage, and treatment. The types of water sources varied by community, as detailed in Table 2. Overall, about half of respondents reported collecting from an unimproved source, from either surface water or unprotected springs. At baseline, 91% of respondents reported collecting water every day and 70.7% reported collecting two to three times per day. The overwhelming majority (97.7%) reported carrying water primarily on their heads and nearly half (47.2%) carried two to three containers of varying types per person per trip. Self-reported time to collect water varied overall, with 38.4% reporting 30 minutes or less round trip and 33.6% reporting between 30 minutes and an hour (Table 2). The community of Mahoue had the largest percentage of respondents (57.1%) reporting less than 30 minutes and Champion had the greatest number of respondents (30.6%) reporting greater than 1 hour. The most commonly reported container used both to carry and store water at baseline was a 20-L bucket, with 98.4% of respondents using a bucket to collect water and 96.4% to store water.

**Table 2:** Frequencies of self-reported round-trip duration of water collection time and type of water source, stratified by community at baseline

	Achen	Anger	Champion	Mahoue	Marotte	Vielot	Total*
Self-reported Time, *n* (%)							
30 minutes or less	33 (36.3)	25 (35.7)	11 (22.4)	52 (57.1)	9 (22.5)	32 (36.8)	168 (38.4)
31 minute to 60 minute	35 (38.5)	26 (37.1)	19 (38.8)	27 (29.7)	17 (42.5)	22 (25.3)	147 (33.6)
> 1 hour	16 (17.6)	13 (18.6)	15 (30.6)	6 (6.6)	9 (22.5)	19 (21.8)	79 (18.0)
Do not know	7 (7.7)	6 (8.6)	4 (8.2)	6 (6.6)	5 (12.5)	14 (16.1)	44 (10.1)
Type of water source *n* (%)†							
Tap stand	73 (80.2)	67 (95.7)	2 (4.1)	41 (45.1)	28 (68.3)	5 (5.7)	226 (51.5)
Spring	9 (9.9)	4 (5.7)	28 (57.1)	52 (57.1)	13 (31.7)	75 (86.2)	181 (41.2)
River	6 (6.6)	1 (1.4)	27 (55.1)	0 (0.0)	0 (0.0)	14 (16.1)	48 (10.9)
Kiosk	5 (5.5)	0 (0.0)	0 (0.0)	0 (0.0)	0 (0.0)	0 (0.0)	6 (1.4)
Open well	2 (2.2)	1 (1.4)	0 (0.0)	0 (0.0)	0 (0.0)	0 (0.0)	3 (0.7)
Pump/cistern	0 (0.0)	0 (0.0)	0 (0.0)	0 (0.0)	1 (2.4)	0 (0.0)	1 (0.2)

* Total includes all communities, including Savan Bourg.

† Multiple responses possible. Percentages do not add up to 100%.

### Self-reported use of packs.

Results varied by proxy variable; however, all trended down over the 6-month period. Overall, self-reported use of the 20-L pack decreased over time from 92.1% at one-month post-distribution to 79.3% at 6 months post-distribution when respondents were asked whether they still used the pack (*P* = 0.164).

**Figure 2.**
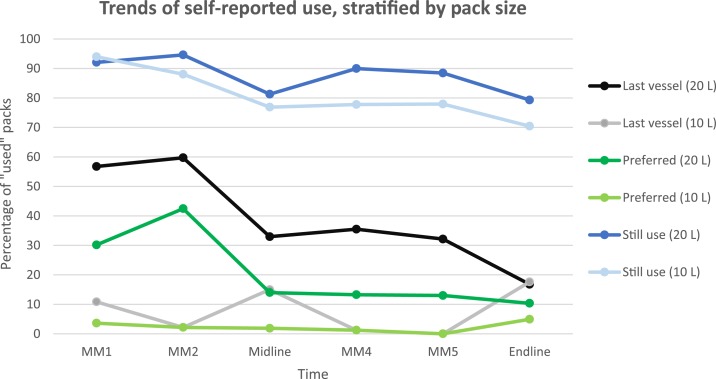
Trends of pack use from November 2014 to May 2015, stratified by pack use and using three proxies for self-reported use including 1) the proportion of respondents reporting to still use packs, 2) the proportion of respondents reporting to have last used packs, and 3) the proportion of respondents who prefer packs. This figure appears in color at www.ajtmh.org.

At 1-month post-distribution, when asked which vessel the respondent used the last time collecting water, 56.7% reported the 20-L pack. This significantly declined to 32.9% at 3 months and 16.8% at 6 months (*P* = 0.0101). The 20-L pack was selected as the preferred container to collect water by 30.2% at 1-month post-distribution. This increased at month 2 but then decreased to less than 15% for months 3 to 6 (*P* = 0.067). Self-reported use varied by community; however, no clear patterns emerged between the three different measures of self-reported use (Figure 2).

Pack recipients continued to prefer strongly the bucket throughout the evaluation, with 79.8% and 82.3% of respondents selecting it as their preferred container at midline and end line, respectively (compared with 13.7% and 10.1% for the 20-L pack at midline and end line). When asked which container (or containers, if they carried multiple) they used the last time they collected water, 80.5% of respondents at midline reported taking a bucket and 41.4% reported taking a one-gallon plastic jug. At end line, 74.5% reported taking a bucket and 18.2% reported taking a plastic jug (compared with 32.3% and 16.4% for the 20-L pack at midline and end line, respectively).

Similar to water collection, most respondents also preferred the bucket for water storage. At midline, 59.6% of respondents reported using a bucket to store drinking water, with 11.4% reporting the 20-L pack. At end line, 58.2% of respondents reported using a bucket to store drinking water, with only 7.0% reporting the 20-L pack for water storage.

### Observed use versus self-reported use.

During the midline and end line water point observations, a total of 1,138 persons were observed collecting water in five of the six communities. Of those observed, 55.6% were female. About half (48.7%) were adults older than 18 years, and 22.7% were children estimated to be less than 10 years of age. More than two-thirds of the people observed were carrying one container. The bucket was most commonly observed, ranging between 41.8% and 55.6% of all observed containers by community. Overall, 6.1% of people observed were carrying a 20-L pack and 5.9% a 10-L pack. Most of the people observed carrying a 20-L pack were males, comprising 85.6% of all those observed with a 20-L pack and 62.7% of those with a 10-L pack. Of those observed carrying the 20-L pack, 63.8% were estimated to be older than the age of 18, 13.0% were estimated to be aged between 10 and 17 years, and 15.9% were estimated to be less than 10 years of age. Of those observed carrying the 10-L pack, 10.5% were estimated to be older than the age of 18 years, 34.3% were estimated to be aged between 10 and 17 years, and 50.7% were estimated to be less than 10 years of age. The age for the remaining 4.5% could not be determined by the water point observers.

**Figure 3.**
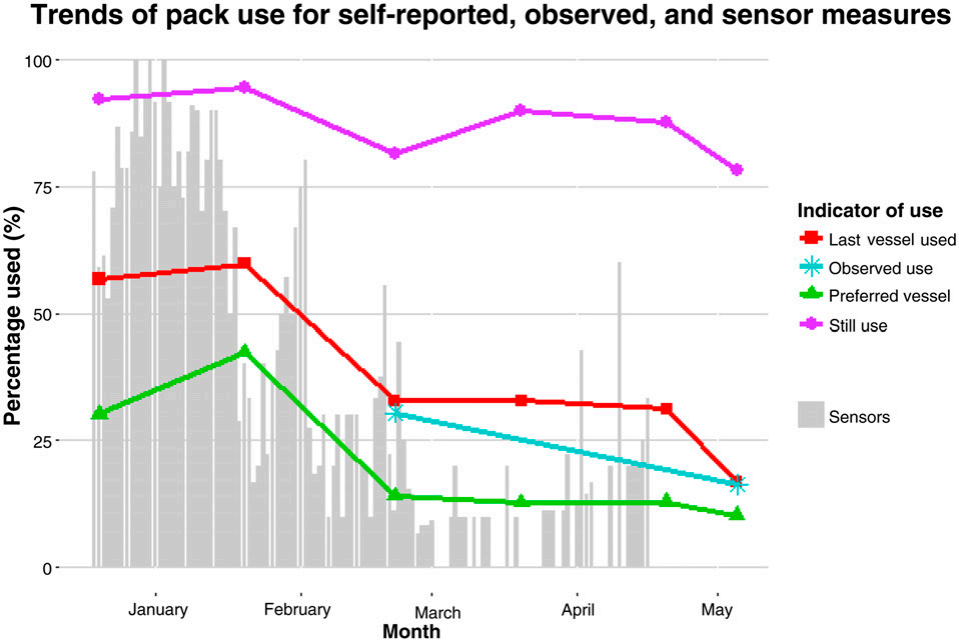
Trends of pack use from December 2014 to May 2015 comparing self-reported, observed, and sensor proxies for use. The self-reported use includes 1) the proportion of respondents reporting to still use packs, 2) the proportion of respondents reporting to have last used packs, and 3) the proportion of respondents who prefer packs. The observed use includes two different time points in February and May of observed proportion of packs (both 20-L and 10-L) of total vessels seen at the water points. NOTE: Self-reported and observed use is combined for 20-L and 10-L packs. Sensor data, which are detailed in supplemental files, are based on a limited number of packs (10–20 packs) compared with self-reported and observed use, which is from a much larger sample size. Vertical bars represent the percent of working sensors, reporting both movement and pressure change suggesting usage. This figure appears in color at www.ajtmh.org.

Observed use of the packs at the source varied between communities (Table 3). Champion saw the highest proportion of individuals observed carrying packs, both 20-L and 10-L. However, the proportion of users in that community still decreased between midline and end line, from 31% to 13% for 20-L packs and from 17% to 8.0% for 10-L packs. In Figure 3, trends of self-reported and observed use are compared with sensor measures, which are presented in detail in supplemental files.

**Table 3:** Comparison of packs observed vs. total people observed at water source in five communities between midline and end line

	Anger (tap stand)	Champion (river)	Mahoue (tap stand)	Marotte (tap stand)	Vielot (river)	Total
Midline						
Individuals with 20-L packs observed	1	24	3	13	5	46
Individuals with 10-L packs observed	0	13	3	18	9	43
Individuals with packs observed/total observations (%)	1/143 (0.7)	37/78 (47.4)	6/156 (3.8)	31/104 (29.8)	14/52 (26.9)	89/533 (16.7)
End line						
Individuals with 20-L packs observed	1	11	0	7	4	23
Individuals with 10-L packs observed	2	7	0	8	7	24
Individuals with packs observed/total observations (%)	3/132 (2.3)	18/88 (20.5)	0/132 (0.0)	15/121 (12.4)	11/132 (8.3)	47/605 (7.8)
Total						
Individuals with 20-L packs observed	2	35	3	20	9	69
Individuals with 10-L packs observed	2	20	3	26	16	67
Individuals with packs observed/total observations (%)	4/275 (1.5)	55/166 (33.1)	6/288 (2.1)	46/225 (20.4)	25/184 (13.6)	136/1,138 (10.2)

Stored water observed in the pack at the household did not change significantly from midline to end line, with 11.0% of packs having water at 3 months and 12.2% at 6 months. Water storage practices varied by community. Mahoue, which had a very small number of observed packs at the water point for transport, saw the largest proportion of households with water in the packs, 21.5% at midline and 26.5% at end line (Table 4), suggesting that the packs in this community were used primarily for water storage in the households but not for water transport.

**Table 4:** Observed households water in the pack at the time of the survey, midline, and end line, stratified by community

Households with water in the pack/total (%)	Achen	Anger	Champion	Mahoue	Marotte	Vielot	Overall
Midline	9/103 (8.7)	5/64 (7.8)	6/51 (11.8)	20/93 (21.5)	4/40 (10.0)	3/76 (4.0)	47/429 (11.0)
End line	19/109 (17.4)	5/64 (7.8)	1/50 (2.0)	22/83 (26.5)	3/41 (7.3)	2/77 (2.6)	52/425 (12.2)

### Factors associated with use.

To identify factors that may influence the uptake of the PackH_2_O, self-reported use was stratified by demographic characteristics and community. Men were significantly more likely than women to have used a pack the last time they went to collect water at end line (OR: 2.59, 95% CI: 1.42–4.70, *P* = 0.002) and more likely than women to prefer a pack to collect water (OR: 4.21, 95% CI: 2.15–8.25, *P* <0.001). Other demographic factors were assessed, including age of respondent and education level, but no significant association was found.

When asked how the 20-L pack could be improved, 65.2% of respondents said that the 20-L pack was too heavy to carry on their backs, 36.9% reported it should be smaller, and 32.8% suggested padding the straps. Other suggestions included converting the pack into another vessel, primarily converting a bucket, so they could carry the pack somewhere other than their back, primarily on the head or in their hands.

## DISCUSSION

We compared self-reported and objective measures of use to assess acceptability of the PackH_2_O in rural Haiti. Both measures indicated a decrease in the overall pack use over 6 months; however, 79.3% of respondents reported to still use the 20-L pack after 6 months. Self-reported measurements may be subject to courtesy, social desirability, and recall biases, and past studies have shown that respondents may exaggerate some hygiene behaviors compared with structured observations.^14^ When asked additional questions about use in this study, such as which container was used the last time for collecting water and preferred container to collect water, results aligned more closely with observed use. At end line, only 16.8% reported to have used the 20-L pack the last time they went to collect water and 10.3% preferred the 20-L pack to collect water (compared with roughly 8% of the people observed at the local water points carrying either a 20-L pack or a 10-L pack and 12% of households visited had water stored in a pack at the time of visit). This suggests that whereas households may exaggerate some behaviors when asked general questions around the use of water, sanitation and hygiene (WASH) services, additional probing during surveys may yield more accurate self-reported data.

Pack use varied between communities, suggesting that social acceptance and distance to water source may influence acceptability. Although five of the six communities were rural and had similar demographics, we observed significant differences in use of the packs for transport and storage among communities. Champion, for which the water source is more remote, had the highest proportion of people coming to the water source with a pack (33% overall from both time points). This is in contrast with that of the community of Mahoue (2% overall from both time points), where the water source is closer and more easily accessible to the community. Self-reported distance to water source was not associated with pack usage; however, the rough, steep terrain and the distance to the water source at Champion may explain higher levels of observed use for transport.

In contrast with the use of the pack for water collection, more than 20% of the households in Mahoue had water in the pack at both the midline and end line visits, the highest proportion of all communities. This suggests that although the pack was not generally being used for collection, beneficiaries preferred the pack for water storage.

The results of this study demonstrate the need for more human-centered design and testing before scaling up WASH interventions, through which communities identify their own challenges and solutions for more convenient, affordable, functional products that they want to use.^15,16^ The majority (82.3%) of respondents still strongly preferred the bucket to the pack after 6 months. Although both containers, a bucket and a 20-L pack, can carry the same load (approximately 44 pounds), carrying loads on the head is the norm in Haiti and therefore may be perceived as physically less exerting than carrying loads on the back.

Although PackH_2_Os were targeted toward females, males were significantly more likely to report to use the pack (as measured by the last vessel used to collect water and preferred vessel). This self-reported use by males was corroborated by observed use, as well; of those people observed carrying a 20-L and 10-L pack at the water point observations, 85.6% and 62.7% were male, respectively. The reasons why men were more likely to prefer the pack than woman and impact of this intervention on lessening the burden of water collection on women and children were outside of the scope of this study; however, more study is warranted of human-centered design of containers that could be targeted toward men for water collection.

Water point observation data suggest that the 10-L pack was used more than the 20-L pack, as the proportion of the 10-L packs distributed that were observed at the water point being used to collect water was twice that of the 20-L packs, both at midline and end line. One of the major complaints of the 20-L pack was that it was too heavy to carry, which could be a reason for more positive feedback for the 10-L pack than the 20-L pack.

No direct input into the introduction and mobilization of the population who received the PackH_2_O was given to implementing partners. It is unclear if additional efforts in introducing the PackH_2_O would have increased uptake and usage.

## LIMITATIONS

The survey data are self-reported and those questions regarding the PackH_2_O are subject to self-reporting bias. Therefore, the actual usage for water transport and storage may be lower than the reported use. The water point observations provided additional evidence regarding the use and nonuse of the packs. However, these were only conducted at two time points, and we did not have any water point observations during the first 3 months when use may have been higher. Age of community members at the water point observations was estimated by the data collectors and not corroborated.

Finally, the distribution of 10-L packs was not completely random at some distribution points and was determined by distribution partners. Because of limited availability, not all households with children received a 10-L pack; however, partners did not purposively distribute to some households over others.

## CONCLUSION

Although reported use was relatively high when asked about use of the pack in general, it was much lower and more aligned with observed use when more specific questions were asked, such as the last time water was collected and the preferred container. In conclusion, the PackH_2_O as an alternative water transport and storage device was not widely accepted or used to collect water in these study communities in rural Haiti. By many, it was considered too heavy, cumbersome, and difficult to carry, as the community is accustomed to carrying loads on the head.

This evaluation was conducted in a relatively stable setting in rural Haiti. We cannot extend these results to other communities or settings in Haiti or elsewhere which is needed, as this type of evaluation provides evidence for identifying effective interventions and improving global health security. The PackH_2_O may be more valuable in an emergency context as it can be prepositioned more easily than classic jerry cans or buckets. Further studies in emergency settings would be a valuable complement to this research and may identify populations, which could benefit from the pack’s design. This product may be more acceptable in communities where carrying loads on backs is already a cultural practice. However, community consultation should be performed before distribution in any context.

## Supplementary Files

Supplemental materials
