# Supplementary material for: Alternative Water Transport and Storage Containers: Assessing Sustained Use of the PackH2O in Rural Haiti
Source: Am J Trop Med Hyg. 2019 Mar 4;100(4):981–7. doi: 10.4269/ajtmh.18-0228 (PMC6447104; doi:10.4269/ajtmh.18-0228)
Supplement: Supplementary file 1 [file tpmd180228.SD1.pdf]

The following are supplemental materials and will be published online only

Supplemental File: Remote Sensor Data

METHODS

A total of 50 sensors were deployed and installed in between the removable plastic liner and the collapsible pack. All consenting households who received an embedded sensor were selected for cross-sectional surveys in addition to those households that were randomly selected. The sensors were equipped with an accelerometer, a global positioning system (GPS) radio, a water pressure transducer, and a Subscriber Identity Module (SIM) card to transmit data directly to an internet database via Global Systems for Mobile communications (GSM) cellular phone technology. The sensors were later reformatted to include a Secure Digital (SD) card to store data locally due to limited cellular phone coverage. The sensors detected movement and pressure differential to indicate use of the pack.

We first compiled data received from the sensors into a daily total movement variable and a sensor-wise normalized pressure variable. All movement values on the days where distribution and reformatting took place were set to missing. Daily values of less than 10 meters were set to zero (no movement) to reflect what was assumed to be movement around the household and not to a water source or other destination. We recorded pressure as a percentage of the maximum reading for that given sensor, in order to normalize results and to account for ambient pressure or temperature differences among the sensors.

Sensor data was recorded for each day between November 17, 2014 and April 17, 2015. Observations after April 17 were set to missing as they were infrequent due to battery failure. We created a proxy variable for use where only the packs with both movement and pressure changes in a single day were considered as having use on that day.

RESULTS

Of the 50 sensors that were deployed, there were 33 sensors with usable data throughout the course of the 6-month evaluation. This was limited to only 3 out of the 6 communities due to lack of cell phone coverage and to the time between November 17, 2014 and April 17, 2015, with a gap in reporting immediately following distribution. On any given day throughout the study, there were between one and 16 sensors that showed

movement and one and 17 sensors that showed a change in pressure. The frequency of daily reports increased after sensor data storage cards were reformatted in mid-December and remained somewhat constant until mid-April.

The proportion of functioning sensors that showed use peaked during the last 2 weeks of month 2, although due to the small number of functional sensors, this is only based on 10 to 18 sensors on any given day. This peak was then followed by another spike in use in the first week of month 3, which corresponded to the time of the midline evaluation. In month 5, sensor reporting dropped off again, as there was no longer field staff available to replace batteries. The figure below shows the proportion of used packs with daily sensor use.

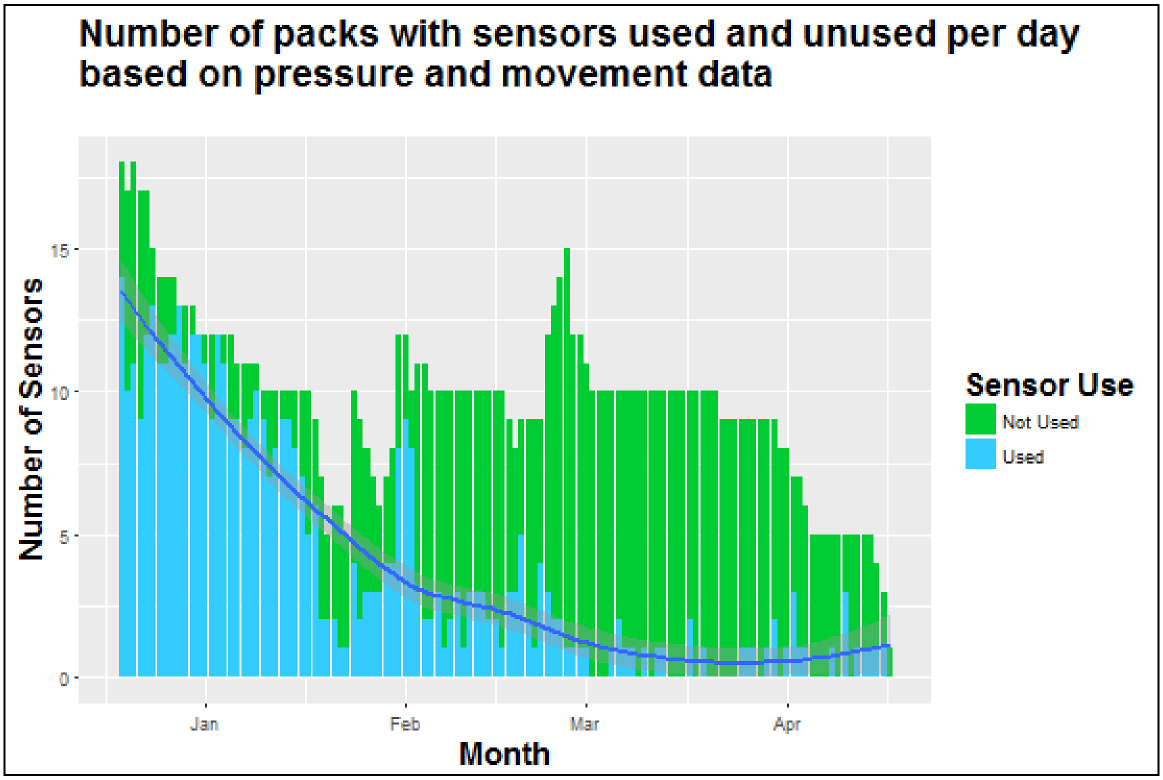

Figure 1: The number of packs with sensors reporting each day with best-fit line, stratified by used and unused from sensor reformatting in mid-December to mid-April, 2015, where assumed use is defined as a daily report showing both movement greater than or equal to 10 meters and a pressure change.

Sensor data showing observed use of packs also declined over time. Although there was no data available for the first month of use after distribution, the proportion of sensors that reported movement and pressure

change data showing use was high during the second month, followed by a precipitous drop in use in third month.

LIMITATIONS

We attempted to document the use of the packs by the addition of the sensors to a sample of pack users. However, the data from sensors is limited to the small number that were functioning throughout the 6-month long evaluation, making it difficult to form definitive conclusions on the objective measure of pack usage. Although the target was to embed 50 live sensors in packs in each community, our data was limited to only 33 in a subset of the communities, and on any given day, sensor data is based on only 18 observations or less. Data from the first month following distribution is very limited, and the frequency of GPS reporting following the second month was much less in order to extend the battery life of the sensors. Because sensors were only recording GPS location every thirty minutes after this reformatting, some trips to the water point could potentially be missed if the house was located close to the source. While daily use was defined as a pack with a sensor reporting at least a 10-meter distance moved and a pressure reading greater than zero, it is difficult to discern whether these cutoffs actually reflect going to the community water source and collecting water.

Additionally, it is unknown as to how the presence of the sensors and the additional weight affected use in households that received them.
